# Prognostic Factors for Adverse Outcomes in Odontogenic Infections Requiring Hospitalization: A Single-Center Retrospective Study in Kraków, Poland

**DOI:** 10.3390/jcm15135120

**Published:** 2026-07-01

**Authors:** Michał Gontarz, Agata Wieczorkiewicz, Andrei Hramyka, Jakub Bargiel, Krzysztof Gąsiorowski, Paweł Szczurowski, Kamil Nelke, Barbara Czopik, Ömer Uranbey, Katarzyna Rusek, Grażyna Wyszyńska-Pawelec

**Affiliations:** 1 Department of Cranio-Maxillofacial Surgery, Jagiellonian University Medical College, 30-688 Cracow, Poland; jakub.bargiel@uj.edu.pl (J.B.); krzysztof.gasiorowski@uj.edu.pl (K.G.); pawel.szczurowski@uj.edu.pl (P.S.); krusek@su.krakow.pl (K.R.); grazyna.wyszynska-pawelec@uj.edu.pl (G.W.-P.); 2 Students’ Scientific Group of the Department of Cranio-Maxillofacial Surgery, Jagiellonian University Medical College, 30-688 Cracow, Poland; agata.wieczorkiewicz@alumni.edu.pl (A.W.); andrei.hramyka@student.uj.edu.pl (A.H.); 3 Maxillo-Facial Surgery Ward, EMC Hospital, Pilczycka 144 Street, 54-144 Wrocław, Poland; kamil.nelke@gmail.com; 4 Private Endodontic Practice PS24, 30-654 Cracow, Poland; bczopik@o2.pl; 5 Department of Oral and Maxillofacial Surgery, Faculty of Dentistry, Aydın Adnan Menderes University, 09010 Aydın, Türkiye; eomeruranbey@gmail.com

**Keywords:** odontogenic infections, deep neck space infections, prognostic factors, phlegmon, treatment outcomes, maxillofacial surgery, mortality

## Abstract

**Background/Objectives**: Odontogenic infections range from localized abscesses to life-threatening deep neck infections and are a frequent cause of emergency admission. We aimed to identify prognostic factors for postoperative complications after their surgical treatment. **Methods**: We retrospectively analyzed 194 adults (59.3% male) treated at the Department of Cranio-Maxillofacial Surgery in Cracow between 2020 and 2025. The primary outcome was any postoperative complication, graded by the Clavien–Dindo classification and dichotomized into minor (grade I–II) and major (grade ≥ III). Prolonged hospitalization and prolonged irrigation (>7 days) were secondary outcomes. Pre-specified main factors (advanced age, diffuse phlegmonous spread, diabetes mellitus) and covariates (sex, maxillary location, systemic disease) were tested by uni- and multivariable logistic regression. **Results**: Comorbidities were present in 69.1%. Complications occurred in 49 patients (25.3%): 19 (9.8%) minor and 30 (15.5%) major, including 12 grade IV intensive-care events and two deaths (grade V). In multivariable analysis, diffuse phlegmonous spread independently predicted any complication (adjusted OR 11.7), major complication (OR 23.4), prolonged hospitalization (OR 5.02) and prolonged irrigation (OR 4.39; all *p* ≤ 0.004). Advancing age independently predicted major complications (OR 1.03 per year, *p* = 0.037). Both fatal cases shared phlegmon, maxillary location and diabetes. **Conclusions**: Diffuse phlegmonous spread was the principal prognostic factor across all adverse outcomes, with advancing age additionally predicting major complications. Because intensive-care admission occurred almost exclusively in phlegmon patients, this association is partly definitional. Early identification of diffuse spread and advanced age may support effective triage.

## 1. Introduction

Odontogenic infections (OIs) represent a significant global health challenge, ranging from localized periapical abscesses to life-threatening deep neck space infections [1,2]. Despite the widespread availability of advanced antibiotic therapies and improved dental hygiene, these infections continue to account for a high volume of emergency hospital admissions and require surgical management [3].

The pathogenesis of OIs is typically polymicrobial, involving a complex synergy between aerobic and anaerobic oropharyngeal flora [4,5]. The infection commonly originates from necrotic pulp or periodontal tissues, disseminating through the alveolar bone into the surrounding soft tissues [4]. The anatomical spread of these infections is dictated by the regional fascial planes, which can facilitate rapid progression into the submandibular, parapharyngeal, and retropharyngeal spaces [6,7]. Ludwig’s angina, a rapidly spreading cellulitis of the submandibular and sublingual spaces, represents the most critical manifestation, often leading to acute airway obstruction and descending necrotizing mediastinitis [8,9].

Effective management of severe OIs requires a multidisciplinary approach focused on early recognition, stabilization, and surgical intervention [3,10]. Contrast-enhanced computed tomography (CECT) is the gold standard for diagnosis, providing important information on the extent of fascial space involvement and potential airway compromise [7,11]. Systemic complications, including Systemic Inflammatory Response Syndrome (SIRS) and sepsis, are major predictors of mortality and often require intensive care unit (ICU) admission [10,12]. The severity of odontogenic infections is multifactorial, with potential contributors including socio-economic factors, delayed patient presentation, and the occurrence of antibiotic-resistant bacterial species. The rising incidence of multidrug-resistant organisms complicates empirical antibiotic selection, necessitating updated treatment protocols [13,14]. Particular caution is required when managing vulnerable populations, such as pregnant patients or the elderly, in whom physiological alterations or multiple comorbidities may mask early symptoms and accelerate progression to SIRS [15,16]. Identifying the key factors that require hospitalization is important for optimizing triage and treatment strategies [3]. Furthermore, the risk of septic progression and the challenges associated with difficult airway management remain persistent concerns for the surgical team, particularly when the infection extends into the deep cervical spaces [9,12].

The aim of this study was to identify prognostic factors for postoperative complications in patients hospitalized and surgically treated for OIs. The primary outcome was the occurrence of any postoperative complication, graded by the Clavien–Dindo classification and dichotomized into minor (grade I–II) and major (grade ≥ III) events: prolonged hospitalization (>7 days) and prolonged abscess irrigation (>7 days) were analyzed as secondary outcomes. Candidate predictors were pre-specified and grouped into main prognostic factors: advanced age, the anatomical extent of infection (diffuse phlegmonous spread), and diabetes mellitus and covariates (sex, body mass index, maxillary location, and the presence of any systemic disease). We hypothesized a priori that the main prognostic factors would be associated with a higher risk of adverse outcomes.

## 2. Materials and Methods

### 2.1. Study Design and Population

This single-center retrospective study includes 194 adult individuals (aged over 15 years) who were admitted to hospital and surgically treated for OIs of the head and neck region. All patients received treatment at the Department of Cranio-Maxillofacial Surgery, University Hospital in Kraków, Poland, between January 2020 and October 2025. Patients were eligible if they had a clinically and radiologically confirmed infection of dental (periapical or periodontal) origin associated with soft-tissue or fascial-space involvement that required hospitalization. This encompassed fascial-space abscesses (buccal, submandibular, perimandibular, submasseteric, temporal, infratemporal and parapharyngeal), Ludwig’s angina and phlegmon. The localized vestibular and submucosal abscesses treated in an outpatient manner (which do not require hospitalization) were excluded from the study. The odontogenic origin was established in every case by clinical examination identifying a causative tooth together with imaging orthopantomography (OPG)/cone beam computed tomography (CBCT) and/or CECT. The exclusion criteria were as follows: insufficient medical documentation, non-odontogenic inflammations, non-infectious pathologies, head and neck abscesses of non-dental origin, abscesses treated in an outpatient manner, and pediatric patients (≤15 years), who were excluded a priori because the etiology and clinical course of odontogenic infection differ in children.

This study was approved by the Institutional Review Board of Jagiellonian University (No 188.0043.1.503.2024). The committee waived the requirement for patient consent due to the retrospective nature of the study and the use of existing medical records, provided that all personal data were kept confidential and anonymized prior to analysis. All necessary data were extracted from electronic health records and included the following: age, systemic diseases, body mass index, symptoms, type and extent of inflammation, causative tooth or teeth, treatment, duration of abscess irrigation, and complications.

### 2.2. Diagnosis and Treatment Protocol

The diagnosis of OIs was based on clinical evaluation (pain, swelling, trismus, dysphagia, fever, and signs of systemic inflammation), identification of a causative tooth, and imaging. OPG or CBCT were performed routinely to identify the dental source, and CECT of the head and neck was obtained whenever deep fascial-space involvement, airway compromise, or systemic deterioration was suspected. The extent of fascial-space involvement and the presence of collections were determined radiologically and confirmed intraoperatively. In every patient, the purulent nature of the infection was verified at operation by the evacuation of pus during incision and drainage. All patients received empirical intravenous antibiotic therapy on admission, subsequently de-escalated to targeted therapy where culture results were available. Surgical source control comprised incision and drainage and/or removal of the causative tooth or teeth. Procedures were performed under local anesthesia for limited abscesses in a single neck space that had ruptured into the subcutaneous tissue. The majority of the remaining infections were treated under general anesthesia for deep, multi-space or airway-threatening infections. The type of anesthesia and the surgical approach (intraoral, extraoral, or combined) therefore reflected infection severity and were recorded as study variables. Postoperative care included continued drainage with irrigation, monitoring of inflammatory parameters, and intensive-care management where indicated.

### 2.3. Severity of Infection Spaces

To account for the heterogeneous severity of OIs, the anatomical extent of disease was used as the principal severity index and was categorized a priori into four groups of increasing infective burden: infection confined to a single fascial space, involvement of two spaces, Ludwig’s angina and diffuse phlegmon involving multiple, non-circumscribed spaces. This division distinguished localized, readily drainable collections from multi-space and life-threatening deep-neck infections, and the number of involved spaces was analyzed as an independent prognostic variable.

### 2.4. Study Variables

The analyzed variables were grouped into three categories. Demographic variables comprised age (divided according to the World Health Organization [WHO] age classification), sex, body mass index (BMI, categorized using WHO criteria), and systemic comorbidities (grouped by affected organ system, with diabetes mellitus analyzed separately given its established immunological relevance). Preoperative (clinical and anatomical) variables comprised the causative tooth and quadrant, the jaw of origin (maxilla, mandible, or both), the number and identity of involved fascial spaces, and the severity stratum defined above. Treatment- and outcome-related (postoperative) variables comprised the type of anaesthesia and surgical approach, the number of extracted teeth, the type of antibiotic therapy, the duration of abscess irrigation, the length of hospitalization and postoperative complications. Inflammatory laboratory markers (e.g., C-reactive protein, procalcitonin, and the neutrophil-to-lymphocyte ratio) and detailed quantitative radiological indices were not consistently available across the whole study population and were therefore not included in the analysis, which is acknowledged as a study limitation. For the outcome analysis, predictors were pre-specified into main prognostic factors (advanced age, diffuse phlegmonous spread, and diabetes mellitus) and covariates (sex, body mass index, maxillary location, and the presence of any systemic disease).

### 2.5. Outcome Definitions

The primary outcome was the occurrence of any postoperative complication. Postoperative complications were graded by severity using the Clavien–Dindo classification into minor and major events. Minor complications (grade I–II) were those requiring no intervention or only pharmacological/bedside management and not threatening life or organ function (e.g., limited mouth opening, conservatively managed surgical-site infection). Major complications (grade ≥ III) were those requiring a reoperation, ICU admission, or resulting in permanent deficit or death (e.g., chronic osteomyelitis, sepsis, permanent nerve injury and death). Prolonged hospitalization (>7 days) and prolonged abscess irrigation (>7 days) were defined as secondary outcomes. Diffuse phlegmonous spread, previously listed among the outcomes, is analyzed in this revision as a prognostic factor.

### 2.6. Statistical Analysis

The statistical analysis was performed using the R Core Team (2024) Program, version 4.5.2 (R Foundation for Statistical Computing, Vienna, Austria). Qualitative variables were compared using the chi-square test with Yates’ continuity correction, 2 × 2 contingency tables, or Fisher’s exact test when the expected cell count assumption was not met. A significance level of 0.05 was applied. Patients were divided into age groups according to the World Health Organization (WHO) classification. WHO classification criteria were applied for BMI categorization. Systemic diseases were categorized into groups according to the affected organ systems. Each prognostic factor was first tested against each outcome in univariable analysis (Fisher’s exact test or the chi-square test with Yates’ correction for categorical variables: univariable logistic regression for age as a continuous variable). Multivariable binary logistic regression models were then constructed for each outcome (any postoperative complication, major complication, prolonged hospitalization, and prolonged irrigation), entering the main prognostic factors (age, phlegmon, diabetes mellitus) together with covariates (sex, maxillary location, systemic disease). Firth’s penalized-likelihood logistic regression was used for outcomes with few events (major complication, prolonged hospitalization) and for in-hospital mortality, to reduce small-sample bias. Given that the number of events per variable was below the conventional threshold of 10 for several outcomes, and that no formal correction for multiple comparisons (e.g., Bonferroni or false-discovery-rate adjustment) was applied, all multivariable estimates were regarded as exploratory and hypothesis-generating. A two-sided *p*-value < 0.05 was considered statistically significant.

## 3. Results

### 3.1. Study Group Characteristics

A total of 194 adult patients with odontogenic abscesses treated between 2020 and 2025 were included in the analysis. Detailed demographic, clinical, and treatment characteristics are summarized in Table 1. The cohort comprised 115 men (59.28%) and 79 women (40.72%). The largest age group comprised patients aged 25–44 years (102; 52.58%). Systemic diseases were reported in 134 patients (69.07%), most commonly metabolic, hematologic, or immunologic disorders (107; 55.15%) and cardiovascular diseases (48; 24.74%). Diabetes was analyzed as a separate condition and was present in 20 patients (10.31%). The majority of patients had normal or slightly elevated BMI: 83 (42.78%) had normal weight, 63 (32.47%) were overweight, and 38 (19.59%) had obesity of varying severity. Most infections involved a single anatomical space (148; 76.29%), while Ludwig’s angina and phlegmon were observed in 11 (5.67%) and 19 (9.79%) cases, respectively. The mandible was the most common site of infection (134; 69.07%). Third molars (78; 40.21%) and second molars (76; 39.18%) were most frequently associated with infection. Surgical treatment was most commonly performed via an extraoral approach under general anaesthesia (130; 67.01%). Postoperative complications occurred in 49 patients (25.3%). When graded by Clavien-Dindo severity, 145 patients (74.74%) had no complication, 19 (9.79%) had a minor (grade I–II) and 30 (15.46%) had a major (grade ≥ III) complication (Table 2). The most frequent minor event was trismus (limited mouth opening, *n* = 12). Major events comprised mechanical ventilation/ICU admission (grade IV, *n* = 12), surgical re-drainage (grade IIIb, *n* = 12), chronic osteomyelitis (grade IIIb, *n* = 5) and death (grade V, *n* = 2). In total, 14 patients required intensive care, of whom two died. The majority of patients received empirical antibiotic therapy (149; 76.80%), and the most common length of hospitalization was 1–3 days (110; 56.70%). The abscess was irrigated for ≤7 days in most patients (147; 75.77%).

### 3.2. Sex

Sex-related differences were observed in infection patterns. Men more frequently had infections involving both the maxilla and mandible, whereas women predominantly presented with mandibular-only infections (*p* = 0.015). First molars were more often affected in men (*p* = 0.015), while no significant sex differences were found for other tooth groups. Men also required more extensive tooth extractions, with three or more teeth removed more frequently than in women (*p* = 0.031). Other parameters, including BMI, the number of involved anatomical spaces, tooth groups other than first molars, dental quadrants, and the duration of abscess irrigation, did not differ significantly between sexes (all *p* > 0.05). Detailed data are presented in Table 3.

### 3.3. Age

The extent of infection and postoperative outcomes were influenced by age. Patients aged 45–59 years most frequently had infections involving a single anatomical space, while two-space involvement was most common in patients aged 25–44 years (*p* = 0.022). Patients aged ≥75 years exhibited a higher prevalence of canine involvement (*p* < 0.001), while a higher frequency of first premolar involvement was observed in patients aged 60–74 years (*p* = 0.005); lateral incisor involvement did not differ significantly between age groups (*p* = 0.107). The present study has demonstrated that individuals of advanced age (≥75 years) are predisposed to a higher risk of chronic osteomyelitis (*p* = 0.055) and mortality (*p* = 0.022). In the 25–44 age group, the predominant cause of purulent inflammation was the impaction of the third molar.

Postoperative complications exhibited variation according to age: the absence of complications was most prevalent in patients ≤ 24 years and least frequent in those aged 60–74 years (*p* = 0.056, a non-significant trend), while mortality occurred in patients aged ≥60 years, specifically within the 60–74 and ≥75 age groups (*p* = 0.022). Subsequent parameters, including BMI, molar involvement, dental quadrants, antibiotic therapy, abscess irrigation duration, and hospitalization length, did not demonstrate significant variation between age groups (all *p* > 0.05). The detailed data can be found in Table 4.

### 3.4. The Number of Involved Anatomical Spaces

The number of involved anatomical spaces was significantly associated with mortality (*p* = 0.021), length of hospitalization (*p* = 0.001), and duration of abscess irrigation (*p* = 0.018). Death occurred exclusively in patients with phlegmon, whereas no deaths were recorded in patients with infections limited to one or two anatomical spaces or in those with Ludwig’s angina. Patients with phlegmon also had the longest hospital stays and the highest proportion of abscess irrigation lasting more than 7 days. No statistically significant differences were found for the number of extracted teeth or for most postoperative complications. Detailed data are presented in Table 5.

### 3.5. Other Factors

No statistically significant associations were observed between procedure type, diabetes, or systemic diseases and postoperative complications, number of affected spaces, tooth extractions, antibiotic therapy, hospitalization duration, or abscess irrigation time (all *p* > 0.05). For procedure type, complication rates were similar across groups (*p* = 0.462), with no deaths reported in any category. In diabetic patients, the proportion of phlegmon appearance was higher (25% vs. 8% in non-diabetics), though not statistically significant (*p* = 0.068), suggesting a potential trend. Patients with systemic diseases showed slightly higher rates of prolonged abscess irrigation (>7 days: 27.6% vs. 16.4%) and some complications, but none reached significance (all *p* > 0.05). These near-threshold trends indicate that larger studies might reveal meaningful associations.

### 3.6. Univariate and Multivariate Logistic Regression Analysis

Each prognostic factor was tested against each outcome univariably (Table 6) and then in multivariable logistic regression models (Table 7) for four outcomes: any postoperative complication, major complication (Clavien–Dindo ≥III), prolonged hospitalization (>7 days), and prolonged abscess irrigation (>7 days). Each model entered the main prognostic factors (age, phlegmon, diabetes mellitus) and covariates (sex, maxillary location, systemic disease). Given only two fatal events, a separate Firth’s penalized logistic regression was applied for mortality (Table 8).

In univariable analysis, diffuse phlegmonous spread was strongly associated with any postoperative complication (OR 11.2, 95% CI 3.8–33.2, *p* < 0.001) and with major complication (OR 20.1, 95% CI 6.8–59.8, *p* < 0.001), reflecting the high rate of intensive-care (grade IV) events among phlegmon patients, and was the principal factor for prolonged hospitalization (OR 6.21, 95% CI 2.19–17.66, *p* = 0.002) and prolonged irrigation (OR 4.08, 95% CI 1.58–10.54, *p* = 0.005). Advanced age was associated with major complications (age ≥ 60 years OR 4.42, 95% CI 1.83–10.69, *p* = 0.001; age per year OR 1.03, *p* = 0.019). Diabetes mellitus showed non-significant trends for major complications (OR 2.68, *p* = 0.094) and prolonged hospitalization (OR 3.19, *p* = 0.057). No covariate (sex, maxillary location, systemic disease, obesity, or number of extractions) was significantly associated with any outcome.

Diffuse phlegmonous spread was the dominant independent predictor of any postoperative complication (aOR 11.73, 95% CI 3.84–35.84, *p* < 0.001) and of major complications (aOR 23.36, 95% CI 7.13–76.52, *p* < 0.001), and remained the only independent predictor of prolonged hospitalization (aOR 5.02, 95% CI 1.66–15.20, *p* = 0.004) and prolonged irrigation (aOR 4.39, 95% CI 1.59–12.14, *p* = 0.004). Advancing age was additionally an independent predictor of major complications (aOR 1.03 per year, 95% CI 1.00–1.06, *p* = 0.037). In the Firth’s penalized mortality model, phlegmon (aOR 14.47; 95% CI 0.77–274; *p* = 0.075) and maxillary location (aOR 9.86; 95% CI 0.55–176; *p* = 0.120) had the largest point estimates but neither was significant. Both fatal cases shared phlegmon, maxillary location and diabetes mellitus. Because mechanical ventilation/ICU admission was classified as a grade IV complication and occurred almost exclusively in phlegmon patients, the very large odds ratios for phlegmon are partly definitional. Together with the small number of events in several outcomes (events per variable below 10), all multivariable estimates remain exploratory and require external validation.

## 4. Discussion

Our retrospective analysis of 194 adult patients identified advanced age (≥60 years), maxillary involvement, and the presence of phlegmon as potential risk factors associated with increased morbidity, and in a very small number of cases mortality in OI. These findings are exploratory in nature and must be interpreted cautiously, particularly regarding mortality, which was observed in only two patients. These findings indicate the importance of early triage, accurate risk stratification and intensive management of high-risk groups to prevent fatal outcomes and reduce hospitalization duration.

In the present study, advanced age was identified as a potential determinant of adverse outcomes, with both fatal cases occurring in patients aged 60 years or older. This observation is in accordance with the findings of the research literature on the physiological sensitivity of the elderly to severe infections [17,18]. Aging is intrinsically associated with immunosenescence, defined as a progressive and multifaceted decline in immune function that impairs the host’s ability to mount an effective and coordinated response against bacterial pathogens [18,19]. This diminished immune capacity frequently results in atypical clinical presentations, delayed diagnoses, and an increased susceptibility to severe sepsis and septic shock in older adults [18,19]. The physiological changes accompanying aging, such as reduced organ reserve, altered drug metabolism, and a higher prevalence of chronic diseases, further complicate the clinical course of severe infections in this demographic [18,19,20]. As noted by Łysenko et al., immune disorders in sepsis represent a profound problem in intensive care, where the delicate balance between pro-inflammatory and anti-inflammatory responses is often disrupted in older adults, leading to multi-organ failure and increased mortality [21]. The SIRS and subsequent compensatory anti-inflammatory response syndrome (CARS) are often dysregulated in the elderly, contributing to a state of persistent inflammation and immunosuppression that predisposes them to secondary infections and poor outcomes [19,20,22]. Furthermore, the high prevalence of multiple comorbidities (69.07%) within our cohort, while not statistically significant in predicting postoperative complications (*p* > 0.05), reflects the complex clinical profile of elderly patients. These comorbidities, such as diabetes, cardiovascular disease, and chronic obstructive pulmonary disease, collectively reduce physiological reserve, making older individuals less resilient to the stress of severe infection and surgical intervention [23,24]. This emphasizes the need for a holistic assessment of geriatric patients presenting with OIs, considering not only the local infection but also their overall systemic health and functional status.

Anatomically, maxillary involvement was associated with mortality compared to mandibular or combined cases in univariate analysis (*p* = 0.02). This association must be interpreted cautiously, given that only two deaths occurred; the finding is of particular significance, given that mandibular infections are more frequently correlated with airway-compromising presentations, such as Ludwig’s angina, which are commonly recognized as the most urgent and potentially life-threatening odontogenic complications [8,12]. However, maxillary infections, particularly those originating from posterior teeth, carry a risk of ascending complications. These include orbital cellulitis, cavernous sinus thrombosis and intracranial abscesses, which are the result of the anatomical relationships of the maxillary sinus, the pterygoid plexus and the rich valveless venous plexus of the midface [25,26,27]. The maxillary sinus, a frequent location for OIs, serves as a direct conduit for bacterial propagation into adjacent vital structures. The thin bone separating the dental apices from the sinus floor, coupled with the potential for direct communication following dental procedures or pathological processes, facilitates the spread of infection [25,26,27]. The spread of infection from the maxillary teeth to the sinonasal mucosa can be profoundly influenced by local environmental factors. Morawska-Kochman et al. demonstrated that the pH value of the sinonasal mucosa can modify bacterial colonization, potentially facilitating the transition from commensal flora to pathogenic biofilms in the presence of an odontogenic source [28]. The pre-existence of bacterial microcolonies on the maxillary sinus ciliary epithelium, even in healthy individuals, suggests a microbial landscape that can be readily exacerbated by purulent odontogenic spread, leading to severe local and systemic complications [29].

The presence of diffuse infection, or phlegmon, was significantly associated with adverse outcomes, including mortality (*p* = 0.021, though based on only two events), prolonged hospital stay (*p* = 0.001), and extended abscess drainage (*p* = 0.018). Unlike localized abscesses that are typically circumscribed by a fibrous capsule and amenable to straightforward surgical drainage, phlegmon represents a non-circumscribed, rapidly spreading inflammatory process that infiltrates multiple anatomical spaces without clear boundaries [8,12,30]. The diffuse nature of the condition often necessitates extensive debridement, multiple surgical interventions, and prolonged antibiotic therapy in order to control the infection [8,12,30,31]. The observed significant sex-related differences, with males presenting more frequent bimaxillary involvement (*p* = 0.015) and requiring a greater number of tooth extractions per patient (*p* = 0.031), warrant further investigation. These discrepancies may be a consequence of socio-cultural factors that impact health-seeking behaviors, resulting in delays in dental care among male patients and, consequently, more extensive disease at the time of presentation [12,32]. Such delays can allow infections to progress from localized processes to diffuse phlegmonous spread, further complicating treatment and worsening prognosis [8,12].

The microbiological profile of these infections is known to have a significant influence on the clinical course of the disease. OIs are typically polymicrobial, involving both aerobic and anaerobic bacteria [31]. The presence of virulent or antibiotic-resistant strains has been demonstrated to promote rapid spread and complicate treatment, necessitating broad-spectrum and subsequently culture-guided therapy [31,33,34]. The rise of antimicrobial resistance poses a significant challenge to empirical management, underscoring the necessity for timely microbiological diagnostics. The progression of the disease from a localized abscess to diffuse phlegmon is driven by microbial synergy, in which aerobic bacteria reduce oxygen tension, thereby facilitating the growth of virulent anaerobes. This process accelerates tissue destruction and may reduce antimicrobial efficacy, emphasizing the importance of prompt surgical source control [33,34,35].

The clinical spectrum of OIs requires special consideration in vulnerable populations. Immunocompromised patients (e.g., those with HIV/AIDS, transplant recipients, or individuals undergoing chemotherapy) are at increased risk of severe and rapidly progressing infections due to impaired immune responses [24,36,37]. Similarly, uncontrolled systemic conditions such as diabetes mellitus predispose patients to worse outcomes by compromising immunity, wound healing, and antibiotic efficacy [24,32]. The present study demonstrated that diabetes mellitus may have a predisposing effect on the development of phlegmon. However, this association did not reach statistical significance (*p* = 0.068). A similar tendency is observed in pregnant patients, in whom physiological changes may obscure disease severity and promote rapid spread, underscoring the need for multidisciplinary care [16,38].

Furthermore, socioeconomic factors, such as access to dental care, health literacy, and financial constraints, significantly impact the presentation and severity of OIs. Patients from underserved populations frequently present at more advanced stages of disease due to delayed access to routine dental services, allowing otherwise manageable conditions to progress into potentially life-threatening infections [12,39,40]. Addressing these disparities remains essential for improving overall public health outcomes. The timing of surgical intervention is closely linked to these socioeconomic determinants. Delayed presentation often results in patients being admitted to maxillofacial units with already advanced OIs, thereby reducing the effectiveness of conservative management and necessitating more aggressive surgical intervention, including extensive debridement [40,41].

To further enhance risk stratification, the integration of systemic inflammatory biomarkers into routine clinical assessment holds considerable promise. Markers such as CRP, procalcitonin, and the NLR have been increasingly recognized for their utility in predicting the severity and clinical course of deep neck space infections [42,43]. Elevated levels of these biomarkers at admission may serve as objective indicators of systemic involvement and impending complications, thereby supporting the early implementation of more aggressive therapeutic strategies, as well as enabling the assessment of treatment efficacy over time [42]. Future prospective studies should focus on the molecular markers of immunosenescence, the role of the sinonasal microbiome in modulating the severity of maxillary OIs, and the impact of socio-economic factors on delayed presentation. This would refine prognostic models and improve patient outcomes. Furthermore, research into the development of novel diagnostic tools for the early detection of phlegmonous spread and personalized antimicrobial regimens based on patient-specific risk profiles would be beneficial. The integration of artificial intelligence and machine learning algorithms has the potential to enhance the predictive modelling of severe OIs. This enhanced predictive modelling would allow for more precise risk stratification and tailored treatment plans, which would ultimately lead to improved patient care and reduced healthcare costs [44].

## 5. Study Limitation

This study has several limitations that should be acknowledged. The relatively small sample size may limit the generalizability of the findings. This limited sample size is a direct consequence of the single-center nature of the study, which inherently restricts the pool of eligible patients. Furthermore, it is important to note that some patients with OIs do not require hospitalization and are managed in outpatient settings, while other specialized centers also treat these conditions. These factors contribute to the observed sample size and may impact the representativeness of our cohort. The analysis was restricted to adult patients. As a single-center study, the results may reflect local clinical practices and patient characteristics, thereby reducing external validity. Additionally, microbiological swabs and antibiotic therapy were not analyzed; the study focused exclusively on clinical and anatomical factors influencing OIs, so the potential impact of microbial profiles and antimicrobial management could not be assessed. Moreover, multiple statistical comparisons were performed across numerous variables without formal correction for multiplicity (e.g., Bonferroni correction or false discovery rate adjustment), which increases the risk of false-positive findings; all reported associations should therefore be considered exploratory and hypothesis-generating. The number of events per variable (EPV) in the multivariable models was below the conventional threshold of 10 for several outcomes. Specifically, this included major complications (*n* = 30) and prolonged hospitalization (*n* = 22), which may result in model overfitting and inflated odds ratio estimates. Inflammatory biomarkers such as CRP, procalcitonin, and the NLR were not systematically collected, limiting the prognostic depth of the analysis; future studies should incorporate these markers. Finally, and most importantly, mortality-related findings are based on only two fatal cases and must be regarded as strictly exploratory: no reliable statistical inference regarding independent mortality predictors can be drawn from such a small number of events. Firth’s penalized model provides estimates, but the extremely wide confidence intervals reflect the inherent uncertainty, and these results require validation in larger prospective multicenter cohorts.

## 6. Conclusions

Our findings provide evidence that advanced age (≥75 years), maxillary location and diffuse anatomical spread (phlegmon) are primary risk factors associated with adverse outcomes in OIs in this exploratory, single-center analysis. Given that mortality analysis is based on only two fatal cases, these findings must be regarded as hypothesis-generating and require validation in larger prospective cohorts. These variables should be carefully assessed during the initial patient evaluation to enable effective triage and guide therapeutic decision-making. Patients presenting with high-risk features should be prioritized for prompt surgical intervention, aggressive antimicrobial therapy and close clinical monitoring, in order to reduce the risk of life-threatening complications. In this analysis, diffuse phlegmonous spread was identified as the principal predictor of postoperative complications and prolonged treatment, emphasising the necessity for early recognition and intensive management of diffuse infection.

## Figures and Tables

**Table 1:** Baseline demographic, clinical, and treatment characteristics of the study cohort.

	Characteristic	Total Number of Patients (N = 194)
Sex	Male	115 (59.28%)
	Female	79 (40.72%)
	15–24 years	30 (15.46%)
	25–44 years	102 (52.58%)
	45–59 years	32 (16.49%)
	60–74 years	19 (9.79%)
	≥75 years	11 (5.67%)
Systemic diseases *	Metabolic/hematologic/immunologic	107 (55.15%)
	Cardiovascular	48 (24.74%)
	Neurological/psychiatric	23 (11.86%)
	Endocrine/genetic	14 (7.22%)
	Genitourinary	12 (6.19%)
	Respiratory	10 (5.15%)
	Musculoskeletal	9 (4.64%)
	Gastrointestinal/liver	8 (4.12%)
	Connective tissue	1 (0.52%)
	Special senses	1 (0.52%)
	None	60 (30.93%)
Diabetes	Present	20 (10.31%)
	Absent	173 (89.18%)
	No response	1 (0.52%)
BMI	Underweight (<18.5)	10 (5.15%)
	Normal weight (18.5–24.9)	83 (42.78%)
	Overweight (25–29.9)	63 (32.47%)
	Obesity class I (30–34.9)	22 (11.34%)
	Obesity class II (35–39.9)	10 (5.15%)
	Obesity class III (≥40)	6 (3.09%)
Involved spaces	One	148 (76.29%)
	Two	16 (8.25%)
	Ludwig’s angina	11 (5.67%)
	Phlegmon	19 (9.79%)
Procedure type	Intraoral GA	16 (8.25%)
	Extraoral GA	130 (67.01%)
	Intraoral LA	24 (12.37%)
	Extraoral LA	17 (8.76%)
	Endoscopic	2 (1.03%)
	Intraoral GA + endoscopic	2 (1.03%)
	Intraoral GA + extraoral GA	3 (1.55%)
Tooth group *	Central incisors	15 (7.73%)
	Lateral incisors	17 (8.76%)
	Canines	28 (14.43%)
	First premolars	27 (13.92%)
	Second premolars	35 (18.04%)
	First molars	56 (28.87%)
	Second molars	76 (39.18%)
	Third molars	78 (40.21%)
	Fourth molars	1 (0.52%)
	Deciduous molars (I)	0 (0.00%)
	Deciduous molars (II)	1 (0.52%)
Dental quadrant *	Upper right	39 (20.10%)
	Upper left	26 (13.40%)
	Lower left	87 (44.85%)
	Lower right	88 (45.36%)
Jaw location	Maxilla	27 (13.92%)
	Mandible	134 (69.07%)
	Both	28 (14.43%)
	No response	5 (2.58%)
Extracted teeth	None	13 (6.70%)
	1	79 (40.72%)
	2	64 (32.99%)
	≥3	38 (19.59%)
Postoperative complications *	None	145 (74.74%)
	ICU admission/mechanical ventilation	14 (7.22%)
	Limited mouth opening	12 (6.19%)
	Reoperation	12 (6.19%)
	Marginal mandibular nerve injury	7 (3.61%)
	Chronic osteomyelitis	5 (2.58%)
	Death	2 (1.03%)
	Surgical site infection	2 (1.03%)
	Chronic sinusitis	1 (0.52%)
	Inferior alveolar nerve injury	1 (0.52%)
Antibiotic therapy	None	1 (0.52%)
	Empirical	149 (76.80%)
	Targeted	44 (22.68%)
Hospital stay	None	2 (1.03%)
	1–3 days	110 (56.70%)
	4–7 days	60 (30.93%)
	>7 days	22 (11.34%)
Abscess irrigation duration	≤7 days	147 (75.77%)
	>7 days	47 (24.23%)

* Multiple choice allowed; percentages may not sum to 100%. GA—general anesthesia; LA—local anesthesia.

**Table 2:** Postoperative complications graded by the Clavien–Dindo classification.

Clavien–Dindo Grade	Severity Category	Complication (Event)	*n*
Grade I	Minor	Limited mouth opening (trismus)	12
Grade I	Minor	Marginal mandibular nerve injury	7
Grade I	Minor	Inferior alveolar nerve injury	1
Grade II	Minor	Surgical site infection (conservative)	2
Grade II	Minor	Chronic sinusitis	1
Grade IIIb	Major	Reoperation (surgical re-drainage)	12
Grade IIIb	Major	Chronic osteomyelitis	5
Grade IV	Major	Mechanical ventilation/ICU admission (survived)	12
Grade V	Major	Death	2

Patient-level dichotomy: no complication 145 (74.74%), minor (grade I–II) 19 (9.79%), major (grade ≥ III) 30 (15.46%). A total of 54 complication events occurred in 49 patients, as some patients experienced more than one event. Fourteen patients required intensive care with mechanical ventilation; in two of them, this was part of a fatal course (grade V), and the remaining twelve were classified as grade IV.

**Table 3:** Comparison of infection sites, tooth involvement, and extractions by sex.

	Characteristic	Male (N = 115)	Female (N = 79)	*p* Value
BMI	Underweight (<18.5)	3 (2.61%)	7 (8.86%)	*p* = 0.221
	Normal weight (18.5–24.9)	55 (47.83%)	28 (35.44%)	
	Overweight (25–29.9)	37 (32.17%)	26 (32.91%)	
	Obesity class I (30–34.9)	13 (11.30%)	9 (11.39%)	
	Obesity class II (35–39.9)	4 (3.48%)	6 (7.59%)	
	Obesity class III (≥40)	3 (2.61%)	3 (3.80%)	
Involved spaces	One	85 (73.91%)	63 (79.75%)	*p* = 0.668
	Two	11 (9.57%)	5 (6.33%)	
	Ludwig’s angina	6 (5.22%)	5 (6.33%)	
	Phlegmon	13 (11.30%)	6 (7.59%)	
Tooth group **	Central incisors	10 (8.70%)	5 (6.33%)	*p* = 0.597
	Lateral incisors	10 (8.70%)	7 (8.86%)	*p* = 1.000
	Canines	15 (13.04%)	13 (16.46%)	*p* = 0.537
	First premolars	16 (13.91%)	11 (13.92%)	*p* = 1.000
	Second premolars	26 (22.61%)	9 (11.39%)	*p* = 0.057
	First molars	41 (35.65%)	15 (18.99%)	*p* = 0.015 *
	Second molars	52 (45.22%)	24 (30.38%)	*p* = 0.051
	Third molars	46 (40.00%)	32 (40.51%)	*p* = 1.000
	Fourth molars	1 (0.87%)	0 (0.00%)	*p* = 1.000
	Deciduous molars (I)	0 (0.00%)	0 (0.00%)	*p* = 1.000
	Deciduous molars (II)	1 (0.87%)	0 (0.00%)	*p* = 1.000
Dental quadrant **	Upper right	29 (25.22%)	10 (12.66%)	*p* = 0.044 *
	Upper left	20 (17.39%)	6 (7.59%)	*p* = 0.055
	Lower left	51 (44.35%)	36 (45.57%)	*p* = 0.884
	Lower right	51 (44.35%)	37 (46.84%)	*p* = 0.770
Jaw location	Maxilla	17 (14.78%)	10 (12.66%)	*p* = 0.015 *
	Mandible	71 (61.74%)	63 (79.75%)	
	Both	23 (20.00%)	5 (6.33%)	
	No response	4 (3.48%)	1 (1.27%)	
Extracted teeth	None	9 (7.83%)	4 (5.06%)	*p* = 0.031 *
	1	42 (36.52%)	37 (46.84%)	
	2	34 (29.57%)	30 (37.97%)	
	≥3	30 (26.09%)	8 (10.13%)	
Abscess irrigation duration	≤7 days	86 (74.78%)	61 (77.22%)	*p* = 0.736
	>7 days	29 (25.22%)	18 (22.78%)	

*p*—chi-square test or Fisher’s exact test. * Statistically significant difference (*p* < 0.05). ** Multiple selections possible—percentages do not sum to 100%.

**Table 4:** Patient characteristics, anatomical involvement, and outcomes by age group.

	Characteristic	≤24 Years (N = 30)	25–44 Years (N = 102)	45–59 Years (N = 32)	60–74 Years (N = 19)	≥75 Years (N = 11)	*p* Value
BMI	Underweight (<18.5)	3 (10.00%)	6 (5.88%)	0 (0.00%)	0 (0.00%)	1 (9.09%)	*p* = 0.132
	Normal weight (18.5–24.9)	16 (53.33%)	47 (46.08%)	8 (25.00%)	7 (36.84%)	5 (45.45%)	
	Overweight (25–29.9)	7 (23.33%)	36 (35.29%)	13 (40.62%)	4 (21.05%)	3 (27.27%)	
	Obesity class I (30–34.9)	1 (3.33%)	7 (6.86%)	6 (18.75%)	6 (31.58%)	2 (18.18%)	
	Obesity class II (35–39.9)	2 (6.67%)	4 (3.92%)	3 (9.38%)	1 (5.26%)	0 (0.00%)	
	Obesity class III (≥40)	1 (3.33%)	2 (1.96%)	2 (6.25%)	1 (5.26%)	0 (0.00%)	
Involved spaces	One	23 (76.67%)	71 (69.61%)	32 (100.00%)	13 (68.42%)	9 (81.82%)	*p* = 0.022 *
	Two	0 (0.00%)	15 (14.71%)	0 (0.00%)	1 (5.26%)	0 (0.00%)	
	Ludwig’s angina	3 (10.00%)	6 (5.88%)	0 (0.00%)	2 (10.53%)	0 (0.00%)	
	Phlegmon	4 (13.33%)	10 (9.80%)	0 (0.00%)	3 (15.79%)	2 (18.18%)	
Tooth group **	Central incisors	0 (0.00%)	7 (6.86%)	3 (9.38%)	4 (21.05%)	1 (9.09%)	*p* = 0.112
	Lateral incisors	0 (0.00%)	7 (6.86%)	5 (15.62%)	3 (15.79%)	2 (18.18%)	*p* = 0.107
	Canines	0 (0.00%)	8 (7.84%)	7 (21.88%)	8 (42.11%)	5 (45.45%)	*p* < 0.001 *
	First premolars	0 (0.00%)	15 (14.71%)	5 (15.62%)	7 (36.84%)	0 (0.00%)	*p* = 0.005 *
	Second premolars	4 (13.33%)	18 (17.65%)	8 (25.00%)	4 (21.05%)	1 (9.09%)	*p* = 0.695
	First molars	11 (36.67%)	32 (31.37%)	9 (28.12%)	1 (5.26%)	3 (27.27%)	*p* = 0.173
	Second molars	15 (50.00%)	41 (40.20%)	12 (37.50%)	5 (26.32%)	3 (27.27%)	*p* = 0.473
	Third molars	8 (26.67%)	49 (48.04%)	14 (43.75%)	4 (21.05%)	3 (27.27%)	*p* = 0.068
	Fourth molars	0 (0.00%)	1 (0.98%)	0 (0.00%)	0 (0.00%)	0 (0.00%)	*p* = 0.924
	Deciduous molars (I)	0 (0.00%)	0 (0.00%)	0 (0.00%)	0 (0.00%)	0 (0.00%)	*p* = 1.000
	Deciduous molars (II)	0 (0.00%)	1 (0.98%)	0 (0.00%)	0 (0.00%)	0 (0.00%)	*p* = 0.924
Dental quadrant **	Upper right	2 (6.67%)	24 (23.53%)	7 (21.88%)	3 (15.79%)	3 (27.27%)	*p* = 0.314
	Upper left	2 (6.67%)	15 (14.71%)	7 (21.88%)	0 (0.00%)	2 (18.18%)	*p* = 0.167
	Lower left	16 (53.33%)	42 (41.18%)	15 (46.88%)	10 (52.63%)	4 (36.36%)	*p* = 0.687
	Lower right	13 (43.33%)	44 (43.14%)	16 (50.00%)	12 (63.16%)	3 (27.27%)	*p* = 0.353
Jaw location	Maxilla	2 (6.67%)	16 (15.69%)	4 (12.50%)	1 (5.26%)	4 (36.36%)	*p* = 0.081
	Mandible	25 (83.33%)	67 (65.69%)	19 (59.38%)	16 (84.21%)	7 (63.64%)	
	Both	2 (6.67%)	16 (15.69%)	8 (25.00%)	2 (10.53%)	0 (0.00%)	
	No response	1 (3.33%)	3 (2.94%)	1 (3.12%)	0 (0.00%)	0 (0.00%)	
Extracted teeth	None	3 (10.00%)	5 (4.90%)	2 (6.25%)	0 (0.00%)	3 (27.27%)	*p* = 0.108
	1	16 (53.33%)	41 (40.20%)	13 (40.62%)	6 (31.58%)	3 (27.27%)	
	2	9 (30.00%)	34 (33.33%)	9 (28.12%)	10 (52.63%)	2 (18.18%)	
	≥3	2 (6.67%)	22 (21.57%)	8 (25.00%)	3 (15.79%)	3 (27.27%)	
Postoperative complications **	None	28 (93.33%)	82 (80.39%)	25 (78.12%)	11 (57.89%)	9 (81.82%)	*p* = 0.056
	Reoperation	0 (0.00%)	7 (6.86%)	2 (6.25%)	3 (15.79%)	0 (0.00%)	*p* = 0.220
	Marginal mandibular nerve injury	0 (0.00%)	3 (2.94%)	4 (12.50%)	0 (0.00%)	0 (0.00%)	*p* = 0.124
	Chronic osteomyelitis	0 (0.00%)	1 (0.98%)	1 (3.12%)	2 (10.53%)	1 (9.09%)	*p* = 0.055
	Limited mouth opening	2 (6.67%)	7 (6.86%)	1 (3.12%)	2 (10.53%)	0 (0.00%)	*p* = 0.810
	Death	0 (0.00%)	0 (0.00%)	0 (0.00%)	1 (5.26%)	1 (9.09%)	*p* = 0.022 *
	Surgical site infection	0 (0.00%)	2 (1.96%)	0 (0.00%)	0 (0.00%)	0 (0.00%)	*p* = 1.000
	Inferior alveolar nerve injury	0 (0.00%)	1 (0.98%)	0 (0.00%)	0 (0.00%)	0 (0.00%)	*p* = 1.000
	Chronic sinusitis	0 (0.00%)	1 (0.98%)	0 (0.00%)	0 (0.00%)	0 (0.00%)	*p* = 1.000
Antibiotic therapy	None	0 (0.00%)	1 (0.98%)	0 (0.00%)	0 (0.00%)	0 (0.00%)	*p* = 0.778
	Empirical	26 (86.67%)	78 (76.47%)	24 (75.00%)	12 (63.16%)	9 (81.82%)	
	Targeted	4 (13.33%)	23 (22.55%)	8 (25.00%)	7 (36.84%)	2 (18.18%)	
Hospital stay	None	0 (0.00%)	2 (1.96%)	0 (0.00%)	0 (0.00%)	0 (0.00%)	*p* = 0.848
	1–3 days	18 (60.00%)	58 (56.86%)	18 (56.25%)	9 (47.37%)	7 (63.64%)	
	4–7 days	10 (33.33%)	31 (30.39%)	11 (34.38%)	5 (26.32%)	3 (27.27%)	
	>7 days	2 (6.67%)	11 (10.78%)	3 (9.38%)	5 (26.32%)	1 (9.09%)	
Abscess irrigation duration	≤7 days	24 (80.00%)	78 (76.47%)	25 (78.12%)	10 (52.63%)	10 (90.91%)	*p* = 0.119
	>7 days	6 (20.00%)	24 (23.53%)	7 (21.88%)	9 (47.37%)	1 (9.09%)	

*p*—chi-square test or Fisher’s exact test. * Statistically significant difference (*p* < 0.05). ** Multiple selections possible—percentages do not sum to 100%.

**Table 5:** Association between the number of involved anatomical spaces and clinical outcomes.

	Characteristic	One Space (N = 148)	Two Spaces (N = 16)	Ludwig’s Angina (N = 11)	Phlegmon (N = 19)	*p* Value
Extracted teeth	None	10 (6.76%)	1 (6.25%)	0 (0.00%)	2 (10.53%)	*p* = 0.540
	1	65 (43.92%)	4 (25.00%)	4 (36.36%)	6 (31.58%)	
	2	49 (33.11%)	7 (43.75%)	3 (27.27%)	5 (26.32%)	
	≥3	24 (16.22%)	4 (25.00%)	4 (36.36%)	6 (31.58%)	
Postoperative complications **	None	119 (80.41%)	12 (75.00%)	10 (90.91%)	14 (73.68%)	*p* = 0.671
	Reoperation	7 (4.73%)	3 (18.75%)	1 (9.09%)	1 (5.26%)	*p* = 0.116
	Marginal mandibular nerve injury	7 (4.73%)	0 (0.00%)	0 (0.00%)	0 (0.00%)	*p* = 1.000
	Chronic osteomyelitis	5 (3.38%)	0 (0.00%)	0 (0.00%)	0 (0.00%)	*p* = 1.000
	Limited mouth opening	9 (6.08%)	1 (6.25%)	0 (0.00%)	2 (10.53%)	*p* = 0.711
	Death	0 (0.00%)	0 (0.00%)	0 (0.00%)	2 (10.53%)	*p* = 0.021 *
	Surgical site infection	2 (1.35%)	0 (0.00%)	0 (0.00%)	0 (0.00%)	*p* = 1.000
	Inferior alveolar nerve injury	1 (0.68%)	0 (0.00%)	0 (0.00%)	0 (0.00%)	*p* = 1.000
	Chronic sinusitis	1 (0.68%)	0 (0.00%)	0 (0.00%)	0 (0.00%)	*p* = 1.000
Hospital stay	None	2 (1.35%)	0 (0.00%)	0 (0.00%)	0 (0.00%)	*p* = 0.001 *
	1–3 days	93 (62.84%)	10 (62.50%)	3 (27.27%)	4 (21.05%)	
	4–7 days	42 (28.38%)	3 (18.75%)	7 (63.64%)	8 (42.11%)	
	>7 days	11 (7.43%)	3 (18.75%)	1 (9.09%)	7 (36.84%)	
Abscess irrigation duration	≤7 days	115 (77.70%)	14 (87.50%)	9 (81.82%)	9 (47.37%)	*p* = 0.018 *
	>7 days	33 (22.30%)	2 (12.50%)	2 (18.18%)	10 (52.63%)	

*p*—chi-square test or Fisher’s exact test. * Statistically significant difference (*p* < 0.05). ** Multiple selections possible—percentages do not sum to 100%.

**Table 6:** Univariable association between each prognostic factor and the four study outcomes.

Predictor	Any Postoperative Complication		Major Complication		Prolonged Hospitalization		Prolonged Irrigation	
	OR (95% CI)	*p*	OR (95% CI)	*p*	OR (95% CI)	*p*	OR (95% CI)	*p*
**Main prognostic factors**								
Age (per year)	1.01 (0.99–1.03)	0.163	1.03 (1.00–1.05)	0.019 *	1.01 (0.99–1.04)	0.346	1.00 (0.99–1.02)	0.650
Age ≥ 60 years	2.29 (1.01–5.18)	0.065	4.42 (1.83–10.69)	0.001 *	2.39 (0.88–6.51)	0.119	1.74 (0.76–3.99)	0.246
Phlegmon	11.20 (3.78–33.19)	<0.001 *	20.14 (6.78–59.84)	<0.001 *	6.21 (2.19–17.66)	0.002 *	4.08 (1.58–10.54)	0.005 *
Diabetes mellitus	1.31 (0.47–3.61)	0.594	2.68 (0.94–7.65)	0.094	3.19 (1.07–9.51)	0.057	1.44 (0.54–3.88)	0.582
**Covariates**								
Male sex	0.89 (0.46–1.71)	0.739	0.75 (0.34–1.64)	0.546	1.49 (0.59–3.75)	0.491	1.13 (0.58–2.21)	0.736
Maxillary location	0.82 (0.31–2.18)	0.814	0.94 (0.30–2.95)	1.000	2.10 (0.73–6.04)	0.201	0.72 (0.27–1.95)	0.629
Systemic disease (any)	1.33 (0.64–2.74)	0.480	1.96 (0.76–5.09)	0.200	0.93 (0.37–2.34)	1.000	1.85 (0.86–3.97)	0.107
Obesity (BMI ≥ 30)	0.90 (0.39–2.06)	1.000	0.79 (0.28–2.23)	0.805	1.31 (0.47–3.66)	0.775	1.38 (0.63–3.02)	0.527
≥3 teeth extracted	1.27 (0.57–2.79)	0.540	2.00 (0.83–4.80)	0.135	1.70 (0.64–4.56)	0.391	1.38 (0.63–3.02)	0.527

OR—odds ratio (univariable logistic regression for age as a continuous variable; Fisher’s exact test or chi-square for categorical predictors); CI—confidence interval. * Statistically significant (*p* < 0.05).

**Table 7:** Multivariable logistic regression of each prognostic factor against the four study outcomes.

Variable	Any Postoperative Complication (Events = 49)		Major Complication, CD ≥ III (Events = 30)		Prolonged Hospitalization > 7 days (Events = 22)		Prolonged Irrigation > 7 days (Events = 47)	
	aOR (95% CI)	*p*	aOR (95% CI)	*p*	aOR (95% CI)	*p*	aOR (95% CI)	*p*
Age (per year)	1.02 (0.99–1.04)	0.125	1.03 (1.00–1.06)	0.037 *	1.01 (0.98–1.04)	0.660	1.00 (0.98–1.02)	0.985
Phlegmon	11.73 (3.84–35.84)	<0.001 *	23.36 (7.13–76.52)	<0.001 *	5.02 (1.66–15.20)	0.004 *	4.39 (1.59–12.14)	0.004 *
Diabetes mellitus	0.50 (0.13–1.93)	0.317	0.71 (0.16–3.23)	0.662	1.94 (0.43–8.77)	0.387	0.94 (0.27–3.25)	0.924
Male sex	0.80 (0.40–1.62)	0.534	0.60 (0.24–1.46)	0.257	1.44 (0.55–3.72)	0.455	1.10 (0.55–2.20)	0.795
Maxillary location	0.86 (0.30–2.48)	0.783	0.86 (0.21–3.51)	0.836	1.78 (0.56–5.67)	0.329	0.61 (0.20–1.83)	0.377
Systemic disease	1.23 (0.54–2.81)	0.617	1.71 (0.55–5.33)	0.353	0.73 (0.26–2.06)	0.552	2.07 (0.89–4.81)	0.093

Each model entered the pre-specified main prognostic factors (age, phlegmon, diabetes mellitus) together with covariates (sex, maxillary location, systemic disease). CD—The Clavien-Dindo Classification; aOR—adjusted odds ratio; CI—confidence interval. Firth’s penalized-likelihood estimation was applied to major complication and prolonged hospitalization. * Statistically significant (*p* < 0.05).

**Table 8:** Firth’s penalized logistic regression analysis of predictors of mortality.

Variable	aOR (95% CI)	*p*-Value
Age ≥ 75 years	2.65 (0.10–69.1)	0.558
Phlegmon	14.47 (0.77–274)	0.075
Maxillary location	9.86 (0.55–176)	0.120
Diabetes mellitus	4.53 (0.13–158)	0.405

*p*—Wald test based on Firth’s penalized likelihood. Both fatal cases shared identical risk factors: phlegmon, maxillary infection, and diabetes mellitus. Wide confidence intervals reflect the very limited number of events (*n* = 2); results are descriptive only. Reference categories: Age < 75 years; No phlegmon; Mandibular/bilateral location; No diabetes.

## Data Availability

The data were obtained from patients operated on at the Cranio-Maxillo-Facial Surgery Department of Jagiellonian University, Cracow, Poland, and cannot be shared in accordance with the General Data Protection Regulation (EU) 2016/679.

## Abbreviations

The following abbreviations are used in this manuscript:

OI	Odontogenic infection
SIRS	Systemic Inflammatory Response Syndrome
BMI	Body Mass Index
ICU	Intensive care unit
OPG	Orthopantomography
CBCT	Cone beam computed tomography
CECT	Contrast-enhanced computed tomography
CARS	Compensatory anti-inflammatory response syndrome
CRP	C-reactive protein
NLR	Neutrophil-to-lymphocyte ratio
